# Technostress Creators and Inhibitors: Dual Impacts on Nurses' Psychological and Work Outcomes

**DOI:** 10.1002/nop2.70458

**Published:** 2026-03-31

**Authors:** Ebtsam Aly Abou Hashish, Hend Abdou Alnajjar

**Affiliations:** ^1^ College of Nursing—Jeddah, King Saud Bin Abdul‐Aziz University for Health Sciences Jeddah Saudi Arabia; ^2^ King Abdullah International Medical Research Center Jeddah Saudi Arabia; ^3^ Ministry of National Guard Health Affairs Jeddah Saudi Arabia; ^4^ Faculty of Nursing Alexandria University Alexandria Egypt

**Keywords:** burnout, digital health, ICT, job satisfaction, nurses, organisational commitment, Saudi Arabia, technostress, transaction‐based stress model

## Abstract

**Aim:**

As healthcare systems continue to adopt digital technologies, nurses are increasingly exposed to technostress—a form of psychological strain associated with the use of technology. This study aimed to assess the levels of technostress among nurses and examine the direct and buffered relationships between technostress creators, technostress inhibitors and work outcomes including strain, job satisfaction, organisational commitment and continuance commitment.

**Design:**

A descriptive correlational study.

**Methods:**

A convenience sample of 250 nurses was recruited from a Saudi hospital. Data were collected using validated self‐administered questionnaires measuring technostress dimensions and outcomes. Statistical analyses included correlation, regression and structural equation modelling to assess direct and mediated relationships.

**Results:**

Nurses reported moderate levels of technostress creators (2.75 ± 0.61) and technostress inhibitors (3.64 ± 0.59). Technostress creators were negatively associated with job satisfaction and commitment and positively linked to strain, while technostress inhibitors demonstrated the opposite pattern. Structural equation modelling confirmed that technostress creators directly increased strain (*B* = 0.85) and reduced job satisfaction, organisational and continuance commitment (*B* = −0.76 to −0.77). Technostress inhibitors not only positively influenced job satisfaction (*B* = 0.01) and organisational commitment (*B* = 0.02) but also partially mediated the relationships between technostress creators and work outcomes, validating the stress‐buffering hypothesis.

**Conclusion:**

Technostress is a significant challenge in digital healthcare settings, contributing to strain and diminished job engagement. However, supportive mechanisms such as digital training and technical assistance can mitigate these effects, play a protective role and enhance well‐being.

**Implications for the Profession and/or Patient Care:**

Addressing technostress is vital for nurses' well‐being. Health systems should prioritise digital literacy and resilience programs, real time technical support and active nurse participation in digital system design to enhance retention, engagement and quality of patient care.

**Impact:**

This study advances understanding of technology‐related psychological burden in nursing by validating the dual‐pathway mechanism through which technostress affects workforce outcomes. The findings inform evidence‐based interventions for hospital leaders and policymakers to develop supportive digital environments that sustain workforce performance, well‐being and digital resilience in increasingly technology‐driven healthcare settings.

**Reporting Method:**

The study followed the STROBE guidelines for cross‐sectional research.

**Patient or Public Contribution:**

This study did not include patient or public involvement in its design, conduct or reporting.

## 1 Introduction

The rapid advancement of information and communication technologies (ICT) has transformed healthcare by improving efficiency, documentation and patient safety through tools like electronic health records and clinical decision support systems (Golz et al. 2024). In response to regulatory and cost pressures, healthcare institutions continue to adopt these technologies to meet evolving service demands (Abou Hashish, and Alnajjar 2024; Abou Hashish, 2025). While nurses benefit from digital tools in delivering quality care, they also face growing challenges. As primary users of healthcare technologies, nurses must continuously adapt to evolving systems under high‐pressure conditions (Shaban et al. 2025; Abou Hashish, and Alnajjar 2024). This has led to technostress—a psychological strain linked to technology‐related demands such as overload, complexity and uncertainty (Ragu‐Nathan et al. 2008). Califf et al. (2020) describe it as the emotional and physical discomfort resulting from interacting with complex and rapidly evolving digital systems. Nurses continually adapt to new applications and workflows, which can lead to frustration, increased workload and exhaustion (Shaban et al. 2025; Ragu‐Nathan et al. 2008). Given their ongoing digital exposure, it is essential to examine how technostress creators and inhibitors influence nurses' well‐being and work outcomes. This study aims to extend the research and literature on this timely topic.

### 1.1 Background

#### 1.1.1 Theoretical Model

**FIGURE 1:**
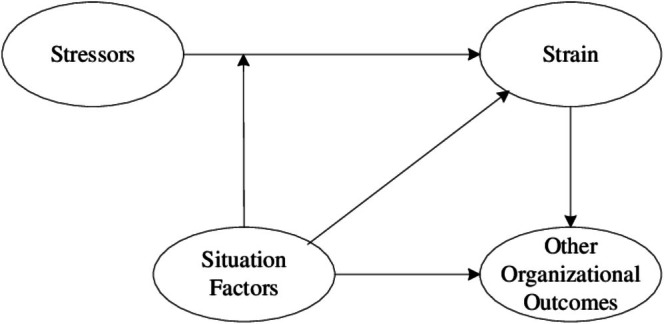
Transaction‐based stress approach.

The transaction‐based approach to stress, developed by Lazarus and Folkman (1984), provides the theoretical foundation for this study. This model conceptualises stress as a dynamic, bidirectional process involving three key stages: (1) primary appraisal (evaluation of demands as threatening or benign), (2) secondary appraisal (evaluation of available coping resources) and (3) stress response outcomes. This model identifies four interrelated components: *Stressors* refer to external demands or conditions in the work environment that may lead to stress. *Situational factors* are organisational mechanisms that can buffer or mitigate the effects of stressors. *Strain* encompasses the psychological, behavioural and physiological outcomes of stress, which may result in decreased job satisfaction, increased absenteeism or turnover. *Organisational outcomes* represent the broader effects of sustained strain on workforce stability and performance. See Figure 1.

Applied to the healthcare context, this framework posits that technological demands (technostress creators) act as environmental stressors that nurses must appraise and respond to, while organisational support mechanisms (technostress inhibitors) represent coping resources that can modify stress responses. The resulting strain and work outcomes (job satisfaction and commitment) represent the culmination of this transactional process. This theoretical perspective has been widely applied in organisational behaviour and workplace stress research, particularly in understanding how individual‐environment interactions shape employee well‐being and performance (Ragu‐Nathan et al. 2008; Tarafdar et al. 2019).

#### 1.1.2 Conceptual Model

Theoretically, the technostress phenomenon can be understood through Lazarus and Folkman's (1984) transaction‐based stress model, which posits that stress arises from the dynamic interplay between environmental demands (stressors) and individual or organisational coping resources (situational factors). In the context of healthcare digitalization, technostress creators represent the demand side of this equation—encompassing technological overload, complexity and uncertainty—while technostress inhibitors represent the resource side, including organisational support, training and participatory involvement in technology implementation (Ragu‐Nathan et al. 2008). Understanding both sides of this equation is essential for developing comprehensive interventions that go beyond merely reducing stressors to actively building digital resilience and adaptive capacity among nursing staff. This dual‐pathway approach examining both the harmful effects of technostress creators and the protective effects of technostress inhibitors provides a more complete picture of how technological demands influence nurses' psychological well‐being and work outcomes.

Henceafter, the transaction‐based approach to stress, developed by Lazarus and Folkman (1984), provides the theoretical foundation for this study. This approach conceptualises stress as a dynamic, bidirectional process involving three key stages: (1) primary appraisal (evaluation of demands as threatening or benign), (2) secondary appraisal (evaluation of available coping resources) and (3) stress response outcomes. Applied to the healthcare context, this framework posits that technological demands (technostress creators) act as environmental stressors that nurses must appraise and respond to, while organisational support mechanisms (technostress inhibitors) represent coping resources that can modify stress responses. The resulting strain and work outcomes (job satisfaction and commitment) represent the culmination of this transactional process.

#### 1.1.3 Technostress Model and Its Application in the Current Study

Based on the theoretical perspectives of the transaction‐based model, Ragu‐Nathan et al. (2008) developed a conceptual model for understanding technological stress. The model integrates the elements of stressors (technostress creators), situational factors (technostress inhibitors), strain and job satisfaction and organisational outcomes such as commitment and continuance commitment (Ragu‐Nathan et al. 2008).

*Stressors* refer to external demands or conditions in the work environment that may lead to stress. In the current study, these are operationalised as technostress creators with five dimensions of technology‐related demands that challenge nurses' adaptive capacity. These include five key dimensions: techno‐overload (working faster and longer due to ICTs), techno‐invasion (blurred boundaries between work and personal life), techno‐complexity (feeling inadequate due to complex systems), techno‐insecurity (fear of job loss due to automation or lack of skills) and techno‐uncertainty (frequent system updates requiring constant learning) (Ragu‐Nathan et al. 2008; Hang et al. 2022).

*Situational factors* are organisational mechanisms that can buffer or mitigate the effects of stressors. In the current study, these correspond to technostress inhibitors, the organisational support structures that provide resources for coping with digital demands operationalised with three dimensions: Literacy facilitation (organisational support for ICT learning and knowledge sharing), technical support provision (availability of timely assistance with ICT problems) and involvement facilitation (participatory users engagement in the introduction and use of new technologies) (Ragu‐Nathan et al. 2008).

*Strain* encompasses the psychological, behavioural and physiological outcomes of stress, which may result in decreased job satisfaction, increased absenteeism or turnover. In the current study measure strain as the immediate psychological distress experienced by nurses in response to technostress. Job satisfaction, conversely, reflects the positive affective evaluation of work experiences when demands are manageable or when adequate support is present. It is a key outcome due to its influence on employee performance and organisational costs (Al‐Ansari and Alshare 2019). Technostress creators are expected to lower job satisfaction, while inhibitors may buffer this effect (Ragu‐Nathan et al. 2008; Hang et al. 2022).

*Organisational outcomes* represent the broader effects of sustained strain on workforce stability and performance. Technostress impacts key organisational outcomes such as These include organisational commitment (emotional attachment to the organisation) and continuance commitment (cost‐based attachment). Organisational commitment reflects an individual's identification with and involvement in their organisation (emotional attachment), while continuance commitment relates to the perceived cost of leaving one's job (intention to remain based on perceived costs of leaving) (Meyer and Allen 1991). Research indicates that higher job satisfaction is positively linked to both forms of commitment. According to the transaction‐based model, technostress inhibitors are expected to strengthen commitment by reducing the negative impact of digital stressors (Ragu‐Nathan et al. 2008; Hang et al. 2022).

*Individual differences*: The model also accounts for individual differences, including age, gender, education level and computer confidence. These factors can shape beliefs about the usefulness and ease of ICT use, thereby affecting levels of technostress (Ragu‐Nathan et al. 2008; Krishnan 2017). This theoretical grounding allows us to examine not only the direct effects of technostress creators and inhibitors on work outcomes but also their interactive effects, consistent with the stress‐buffering hypothesis embedded in the transaction‐based model.

### 1.2 Research Hypotheses

**FIGURE 2:**
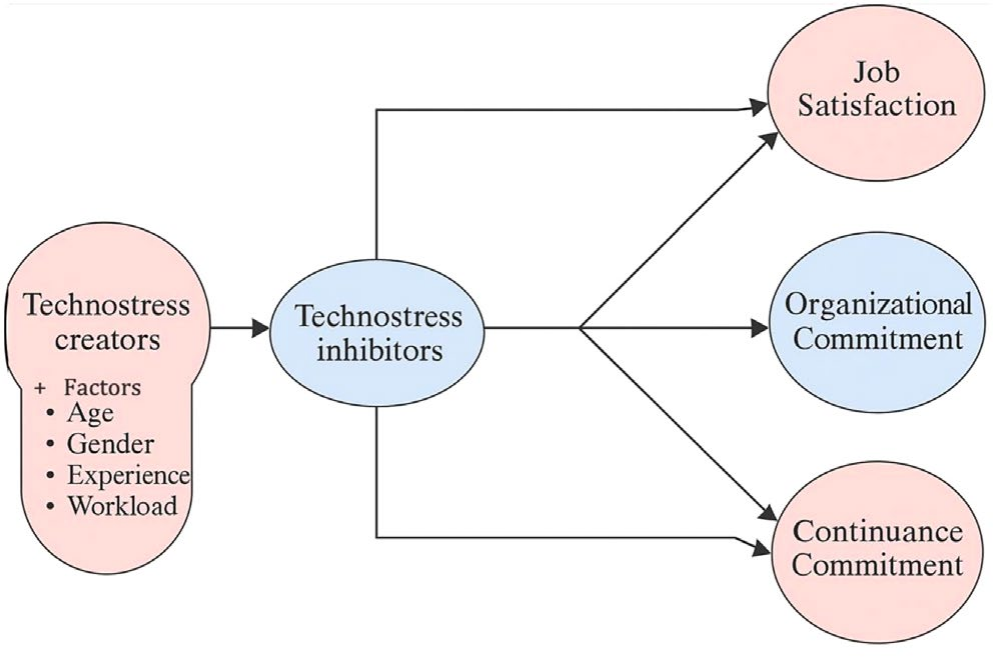
Proposed study framework.

Drawing from Lazarus and Folkman's (1984) transaction‐based model and the technostress framework (Ragu‐Nathan et al. 2008), the researchers expect that technostress creators (stressors) will directly increase strain and reduce positive work outcomes. Conversely, technostress inhibitors (situational factors) should not only have direct positive effects on work outcomes but also serve as protective factors that buffer or attenuate the negative effects of technostress creators. This dual pathway—direct effects and mediating effects—forms the basis of the hypothetical model. See Figure 2.

The following specific directional hypotheses sets are proposed:

### 1.3 Effects of Technostress Creators (Hypothesis Set 1)

*Technostress creators will be positively associated with strain among nurses*.

*Technostress creators will be negatively associated with job satisfaction among nurses*.

*Technostress creators will be negatively associated with organisational commitment among nurses*.

*Technostress creators will be negatively associated with continuance commitment among nurses*.

### 1.4 Effects of Technostress Inhibitors (Hypothesis Set 2)

*Technostress inhibitors will be negatively associated with strain among nurses*.

*Technostress inhibitors will be positively associated with job satisfaction among nurses*.

*Technostress inhibitors will be positively associated with organisational commitment among nurses*.

*Technostress inhibitors will be positively associated with continuance commitment among nurses*.

### 1.5 Mediating Effect of Technostress Inhibitors (Hypothesis Set 3)

*Technostress inhibitors will mediate the relationships between technostress creators and work outcomes among nurses. Specifically, the presence of organisational support mechanisms (inhibitors) will buffer (reduce) the negative effects of technostress creators on strain, job satisfaction, organisational commitment and continuance commitment*.

### 1.6 Significance of the Study

As healthcare systems rapidly embrace digital transformation, nurses are increasingly expected to adapt to evolving technologies in their daily work. While the benefits of information and communication technologies (ICT)—such as enhanced documentation and patient safety—are widely acknowledged, their psychological toll, particularly in the form of technostress, remains underexplored in clinical practice (Hang et al. 2022). Existing studies tend to focus on general stress levels among nurses, with limited attention to how technostress specifically impacts key work outcomes such as job satisfaction, strain and organisational commitment—especially in technology‐intensive hospital settings undergoing accelerated digital implementation. Technostress has been associated with emotional fatigue, job dissatisfaction and diminished well‐being, arising from nurses' interaction with complex and frequently updated digital systems (Golz et al. 2024; Califf et al. 2020). The continual need to master new digital tools adds to cognitive overload and emotional strain, threatening both individual performance and workforce sustainability (Shaban et al. 2025; Abou Hashish and Alnajjar 2025).

This study addresses these gaps by examining both technostress creators and inhibitors within a unified model and assessing their combined impact on nurses' work outcomes. It offers timely evidence to guide policy and practice in fostering digital resilience, mitigating psychological burden and optimising nurse engagement in digitally advanced healthcare environments.

### 1.7 Aim of the Study

This study aimed to: (1) assess the levels of technostress creators and inhibitors among nurses, (2) examine the direct relationships between technostress creators, technostress inhibitors and work outcomes (strain, job satisfaction, organisational commitment and continuance commitment) and (3) investigate whether technostress inhibitors mediate the relationships between technostress creators and work outcomes.

## 2 Methods

### 2.1 Study Design and Setting

A descriptive‐correlational study was conducted at a Saudi hospital, a 750‐bed tertiary facility that uses the Health Information System (BESTCare) with nurses playing a key role in leveraging its features to support digital documentation, clinical decision‐making and patient care (MNGHA‐BESTCare 2024). The study followed the STROBE guidelines.

### 2.2 Sampling and Participants

A convenience sample of nurses from inpatient care units was recruited. Eligible participants were nurses with at least six months of experience. Recently hired or student nurses were excluded. Using the Raosoft calculator, a minimum sample of 191 was determined (5% margin of error, 95% confidence level). Of 400 questionnaires distributed, 250 were completed and included in the analysis. The response rate was 62.5% (250/400).

### 2.3 Data Collection Instrument

Section 1: *Nurses' demographics and work characteristics*, such as age, gender, nationality, educational level, working unit, years of experience, nurse–patient ratio, previous ICT training programs, familiarity with IT systems, number of hours worked per week and extra shifts.

Section 2: *The Technostress Questionnaire*, adapted from Ragu‐Nathan et al. (2008), consists of two domains: the technostress creators domain includes 23 items across five dimensions: techno‐overload (5 items), techno‐invasion (4 items), techno‐complexity (5 items), techno‐insecurity (5 items) and techno‐uncertainty (4 items). Technostress inhibitors consist of 13 items with three dimensions: literacy facilitation (5 items), technical support provision (4 items) and involvement facilitation (4 items).

Section 3: *Technostress outcomes* include strain, job satisfaction, organisational commitment and continuance commitment. Strain was measured using three items adapted from Kasemy et al. (2022). Job satisfaction was assessed using Spector's (1985) three‐item scale, while organisational and continuance commitment were measured using four items each from Meyer and Allen (1997). Responses for Sections 2 and 3 were rated on a 5‐point Likert scale (1 = strongly disagree to 5 = strongly agree), with higher scores reflecting greater perceptions.

### 2.4 Validity and Reliability

The instrument was administered in its original English version. The questionnaire, previously validated (Kasemy et al. 2022; Ragu‐Nathan et al. 2008), showed reliability coefficients ranging from 0.80 to 0.90. Content validity was confirmed through expert review. A pilot study involving 5% of the target sample confirmed the tool's clarity and usability, with no changes needed. Internal consistency was assessed using Cronbach's alpha, with strong reliability reported for the overall scale (*α* = 0.887, *p* ≤ 0.05). Subscale values were: technostress creators (*α* = 0.901), inhibitors (*α* = 0.866), strain (*α* = 0.822), job satisfaction (*α* = 0.884), organisational commitment (*α* = 0.857) and continuance commitment (*α* = 0.841).

### 2.5 Data Collection

Following ethical approval from King Abdullah International Medical Research Center (KAIMRC) and nursing directors, the study questionnaire was distributed in both electronic and paper formats. QR codes linking to the online version were placed at nursing stations. Nurses were individually contacted via email and WhatsApp (with permission) to select their preferred format. After obtaining informed consent, the study's purpose was explained, and clarifications were provided as needed. Data collection occurred over 4 months, from October 2024 to January 2025.

### 2.6 Data Analysis

Data were analysed using SPSS version 25. Of the 256 returned questionnaires, 250 met inclusion criteria and were included in the analysis. Normality was assessed using the Shapiro–Wilk test, confirming suitability for parametric tests.

Descriptive statistics (means, standard deviations, frequencies and percentages) were used to summarise demographic characteristics and study variables. To test Hypotheses 1 and 2 (H1a–d, H2a–d), Pearson correlation coefficients examined bivariate relationships between technostress creators, technostress inhibitors and work outcomes (strain, job satisfaction, organisational commitment and continuance commitment). Multiple linear regression analyses identified significant predictors of each outcome variable. To test Hypothesis 3 (mediation effects), structural equation modelling (SEM) with path analysis was conducted using AMOS. The mediation model examined whether technostress inhibitors mediated the relationships between technostress creators and work outcomes. Model fit was evaluated using multiple indices: Chi‐squared/df ratio (acceptable < 3), Comparative Fit Index (CFI ≥ 0.90), Incremental Fit Index (IFI ≥ 0.90) and Root Mean Square Error of Approximation (RMSEA ≤ 0.08). Direct, indirect and total effects were estimated. Statistical significance was set at *p* ≤ 0.05 for all analyses.

### 2.7 Ethical Considerations

The study received ethical approval from KAIMRC with Institutional Review Board (IRB) approval no. NRJ24/032/8. All procedures followed ethical standards for research involving human participants. Nurses were informed about the study's purpose, risks, benefits and their rights, including voluntary participation and withdrawal without penalty. Written informed consent was obtained, and confidentiality, anonymity and secure data handling were maintained throughout the study.

## 3 Results

### 3.1 Demographic and Work Characteristics

The majority of nurses were female (80.8%) and predominantly non‐Saudi nationals (78.4%). Nearly half (49.2%) were between 30 and < 40 years, with a mean age of 37.15 ± 4.86 years old. Most nurses held a bachelor's degree (68.4%). The largest proportion were working in surgical wards (33.6%), followed by oncology (26.4%). More than half of the nurses had between 1 and 10 years of experience (57.6%), with a mean of 9.62 ± 7.18 years. Most nurses reported caring for 1–4/5 patients (42%), and 79.6% worked ≥ 40 h per week, with a mean of 43.61 ± 16.61 h. Notably, 73.2% reported working extra shifts. With regard to digital literacy and system exposure, over half of the participants (54.4%) had previous ICT training, and 61.6% reported being familiar with the IT system. See Table 1.

**TABLE 1:** Distribution of the studied sample according to their demographic data (*N* = 250).

Demographic characteristics	No.	%
**Gender**		
Male	48	19.2
Female	202	80.8
**Nationality**		
Saudi	54	21.6
Non Saudi	196	78.4
**Age category**		
20–< 30	45	18.0
30–< 40	123	49.2
40–< 50	58	23.2
≥ 50 years	24	9.6
**Mean ± SD**	37.15 ± 4.86	
**Educational level**		
Bachelor level	171	68.4
Diploma	69	27.6
Master	10	4.0
**Working unit**		
Medical ward	46	18.4
Surgical ward	84	33.6
Obstetrics ward	27	10.8
Emergency department	3	1.2
Intensive care unit	24	9.6
Oncology	66	26.4
**Years of experience**		
< 1 year	24	9.6
1–5 years	72	28.8
6–10 years	72	28.8
11–15 years	36	14.4
16–20 years	16	6.4
21 years or more	30	12.0
**Mean ± SD**	9.62 ± 7.18	
**Nurse–patient ratio**		
1–1	60	24.0
1–3	61	24.4
1–4/5	105	42.0
1–> 5	24	9.6
**Number of hours worked per week**		
20–< 40	51	20.4
≥ 40	199	79.6
**Mean ± SD**	43.61 ± 16.61	
**Working extra shifts during the month**		
Yes	183	73.2
No	67	26.8
**Previous information technology (ICT) training**		
Yes	136	54.4
No	114	45.6
**Degree of familiarity with IT system**		
Familiar	154	61.6
Slightly Familiar	81	32.4
Not familiar at all	15	6.0

### 3.2 Perceived Technostress Variables Among Nurses

**FIGURE 3:**
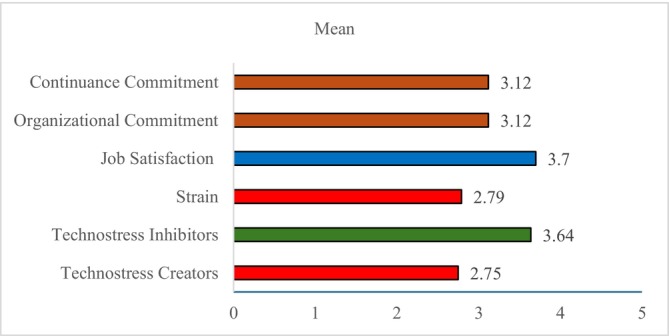
Mean score of overall technostress creators, inhibitors and work outcomes.

Table 2 and Figure 3 show that the overall average score for technostress creators was 2.75 ± 0.61, with a moderate percent score of 43.85%. Among the dimensions, techno‐uncertainty had the highest score (65.25%), while techno‐invasion (31.28%) was the lowest. In contrast, technostress inhibitors had a higher average score of 3.64 ± 0.59 and a percent score of 65.88%, with literacy facilitation (70.32%) and technical support provision (65.78%) rated as the most effective supports. Technostress outcomes had an average score of 3.17 ± 0.46 and a percent score of 54.32%, indicating moderate effects. Job satisfaction was relatively high (67.40%), while strain was lower (44.80%). Organisational and continuance commitment fell near the midpoint, reflecting moderate levels of loyalty and intent to stay.

**TABLE 2:** Perceived technostress variables among nurses.

Variable	Average score	Percent score
**Technostress creators**	2.75 ± 0.61	43.85
Techno‐overload	3.05 ± 0.96	51.30
Techno‐invasion	2.25 ± 0.86	31.28
Techno‐complexity	2.59 ± 0.76	39.78
Techno‐insecurity	2.34 ± 0.73	33.42
Techno‐uncertainty	3.61 ± 0.76	65.25
**Technostress inhibitors**	3.64 ± 0.59	65.88
Literacy facilitation	3.81 ± 0.63	70.32
Technical support provision	3.63 ± 0.69	65.78
Involvement facilitation	3.42 ± 0.70	60.45
**Technostress outcomes**	3.17 ± 0.46	54.32
Strain	2.79 ± 0.85	44.80
Job satisfaction	3.70 ± 0.70	67.40
Organisational commitment	3.12 ± 0.78	53.03
Continuance commitment	3.12 ± 0.70	52.95

### 3.3 Hypothesis Testing: Relationships Among Technostress Creators, Inhibitors and Outcomes

#### 3.3.1 Testing Hypotheses 1 and 2 (Direct Effects)

Correlation analysis examined the bivariate relationships predicted in Hypotheses 1 and 2. Results revealed that technostress creators showed significant negative correlations with job satisfaction (*r* = −0.206, *p* ≤ 0.05), organisational commitment (*r* = −0.214, *p* ≤ 0.05), and continuance commitment (*r* = −0.302, *p* ≤ 0.05), while being positively associated with strain (*r* = 0.277, *p* ≤ 0.05). These findings support Hypotheses H1a, H1b, H1c and H1d.

Conversely, technostress inhibitors demonstrated significant positive correlations with job satisfaction (*r* = 0.154, *p* ≤ 0.05), organisational commitment (*r* = 0.396, *p* ≤ 0.05) and continuance commitment (*r* = 0.349, *p* ≤ 0.05), while showing a strong negative correlation with strain (*r* = −0.885, *p* ≤ 0.05). These findings support Hypotheses H2a, H2b, H2c and H2d. See Table S1 for detailed correlation values.

Multiple linear regression analyses (Table 3) confirmed the significant predictive effects of both technostress creators and inhibitors on overall technostress outcomes. Technostress creators were a significant negative predictor (*B* = −0.34, *p* < 0.001), explaining 38.6% of the variance in technostress outcomes. Technostress inhibitors were a significant positive predictor (*B* = 0.01, *p* < 0.001), explaining 42% of the variance.

**TABLE 3:** Linear regression analysis for the parameters affecting technostress outcomes.

Factors	*B*	*t*	*p*	95% CI		*R* ^2^ (Effect Size)	Adjusted *R* ^2^
				LL	UL		
Technostress creators	−0.34	−9.935	< 0.001*	−0.402	−0.269	0.386	0.383
Technostress inhibitors	0.014	3.818	< 0.001*	0.005	0.016	0.420	0.415

Abbreviations: *B*, Unstandardized Coefficients; CI, Confidence interval; LL, Lower limit; *R* ^2^, Coefficient of determination; *t*, *t*‐test of significance; UL, Upper Limit.

* Statistically significant at *p* ≤ 0.05.

#### 3.3.2 Testing Hypothesis 3 (Mediation Effects)

**FIGURE 4:**
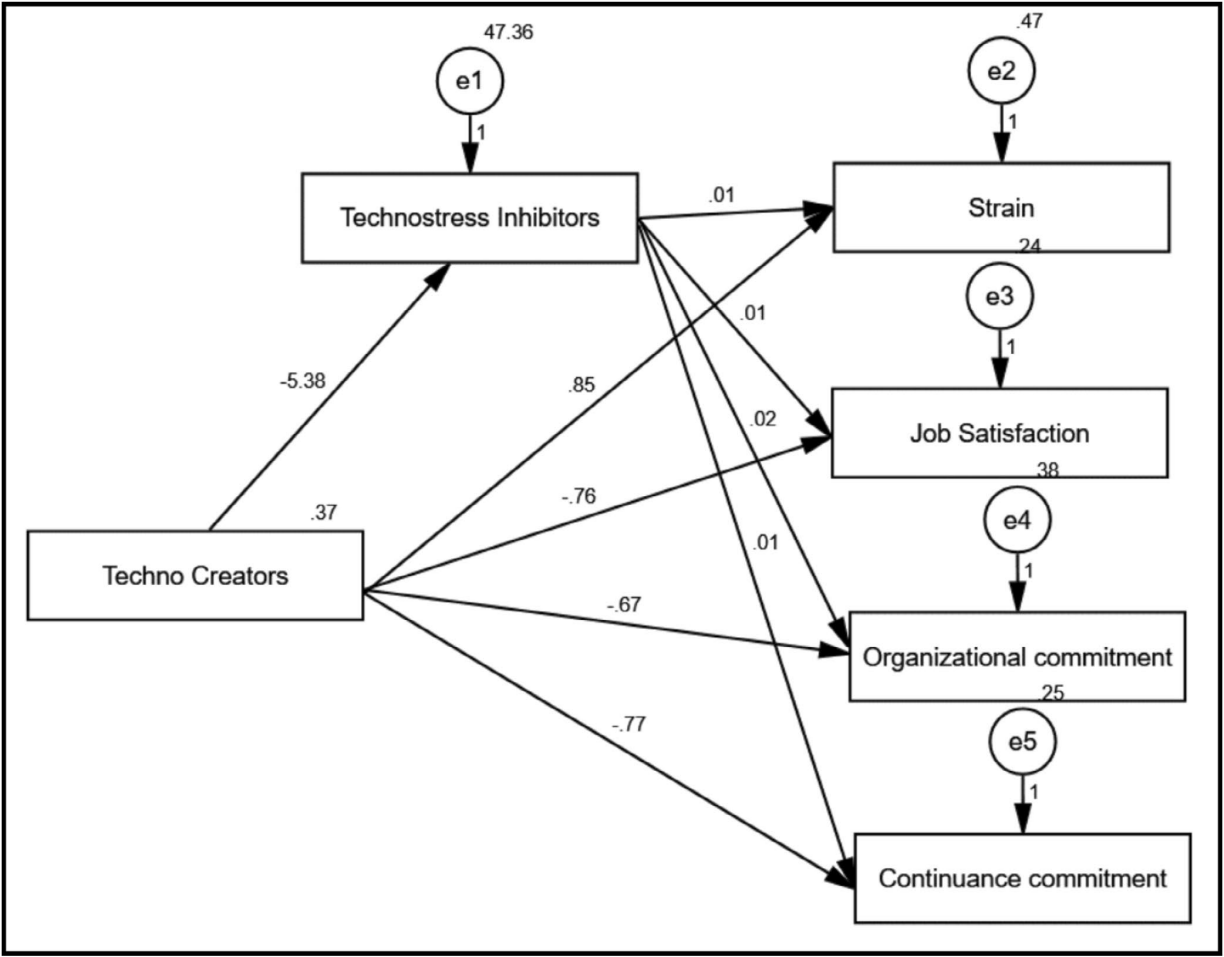
The effect of technostress creators on technostress outcomes mediated by technostress inhibitors.

Structural equation modelling (SEM) with path analysis was conducted to test Hypothesis 3 regarding the mediating role of technostress inhibitors (Table 4 and Figure 4). Results showed that technostress creators had a strong negative direct effect on technostress inhibitors (*B* = −5.38, *p* < 0.001), indicating that higher levels of technostress creators are associated with lower perceptions of organisational support. The direct effects of technostress creators on work outcomes were statistically significant. Specifically, technostress creators were associated with increased strain (*B* = 0.85, *p* < 0.001) and decreased job satisfaction (*B* = −0.76, *p* < 0.001), organisational commitment (*B* = −0.67, *p* < 0.001) and continuance commitment (*B* = −0.77, *p* < 0.001).

**TABLE 4:** The total, direct and indirect effect of technostress creators on technostress outcomes mediating by Technostress Inhibitors.

Variables	Total effect	Direct effect	Indirect effect	95% CI		*t*–statistics	*p*
				LL	UL		
Technostress inhibitors ← Technostress creators	−5.38	−5.38		−5.879	−4.634	−7.510	< 0.001*
Strain ← Technostress inhibitors	0.01	0.01		−0.005	0.020	1.233	0.217
Job satisfaction ← Technostress inhibitors	0.01	0.01		0.002	0.020	2.448	0.014*
Organisational Commitment ← Technostress inhibitors	0.02	0.02		0.005	0.027	2.867	0.004*
Continuance commitment ← Technostress inhibitors	0.01	0.01		−0.003	0.015	1.346	0.178
Strain ← Technostress creators	0.81	0.85	0.01	0.669	0.951	10.816	< 0.001*
Job satisfaction ← Technostress creators	−0.82	−0.76	−0.01	−0.917	−0.714	−13.383	< 0.001*
Organisational commitment ← Technostress creators	−0.76	−0.67	−0.01	−0.888	−0.631	−9.504	< 0.001*
Continuance commitment ← Technostress creators	−0.80	−0.77	−0.01	−0.904	−0.696	−13.230	< 0.001*

*Note:* Model fit parameters: CFI; IFI; RMSEA (0.992; 0.999; 0.071), Model *X* ^2^/1.070.

Abbreviations: CFI, Comparative Fit Index; IFI, Incremental Fit Index; RMSEA, Root Mean Square Error of Approximation.

* *p* = 0.378.

Regarding mediation effects, technostress inhibitors significantly and positively influenced job satisfaction (*B* = 0.01, *p* < 0.001) and organisational commitment (*B* = 0.02, *p* < 0.001), demonstrating a buffering effect. The model demonstrated excellent fit: *χ* ^2^/df = 1.070, *p* = 0.378; CFI = 0.992; IFI = 0.999; RMSEA = 0.071. These findings provide support for Hypothesis 3, indicating that technostress inhibitors partially mediate the relationships between technostress creators and work outcomes.

### 3.4 Demographic Characteristics and Technostress Variables

Significant differences were found across demographic groups. Female nurses and Saudi nationals reported higher technostress creator scores (*p* < 0.001). Younger nurses (20–< 30 years) had higher creator scores and lower inhibitor scores (*p* = 0.002; *p* = 0.047). Nurses with a master's degree showed the highest creator scores (*p* = 0.014). ICU nurses reported higher technostress creators than other units (*p* = 0.001). Nurses with prior ICT training had higher technostress creators (*p* = 0.002) but lower outcome scores (*p* < 0.001). Those unfamiliar with IT systems showed higher creators (*p* = 0.027) and lower inhibitors (*p* < 0.001). Working fewer than 40 h per week was associated with higher inhibitor perceptions (*p* < 0.001). See Table 5.

**TABLE 5:** Relationship between demographic data and study variables.

Demographic characteristics	Technostress creators	Technostress inhibitors	Technostress outcomes
	Mean ± SD	Mean ± SD	Mean ± SD
**Gender**			
Male	57.31 ± 10.21	48.00 ± 5.28	56.80 ± 7.49
Female	64.78 ± 14.44	47.08 ± 8.10	54.01 ± 9.98
***t* (*p*)**	4.170 (< 0.001)*	0.963 (0.338)	1.822 (0.070)
**Nationality**			
Saudi	69.33 ± 15.58	46.44 ± 8.03	52.60 ± 11.69
Non Saudi	61.69 ± 13.13	47.48 ± 7.53	55.08 ± 8.90
***t* (*p*)**	3.631 (< 0.001)*	0.886 (0.377)	1.444 (0.153)
**Age category**			
20–< 30	70.40 ± 12.85	44.53 ± 7.58	51.84 ± 8.22
30–< 40	62.32 ± 13.93	48.27 ± 6.85	55.64 ± 10.02
40–< 50	60.29 ± 13.80	47.19 ± 8.43	53.74 ± 8.44
≥ 50 years	62.75 ± 13.56	47.38 ± 8.73	55.92 ± 11.64
***F* (*p*)**	5.199 (0.002)*	2.684 (0.047)*	2.057 (0.107)
**Educational level**			
Bachelor level	62.16 ± 13.18	46.96 ± 8.05	54.61 ± 9.88
Diploma	64.57 ± 14.04	48.04 ± 6.98	54.57 ± 8.15
Master	73.33 ± 22.22	46.33 ± 3.50	53.99 ± 14.84
***F* (*p*)**	3.607 (0.014)*	0.497 (0.685)	0.223 (0.880)
**Working unit**			
Medical ward	65.28 ± 13.73	45.72 ± 6.81	54.10 ± 8.41
Surgical ward	58.89 ± 14.62	48.29 ± 9.09	56.47 ± 9.47
Obstetrics ward	68.67 ± 12.18	45.33 ± 5.15	51.43 ± 7.11
Emergency department	58.00 ± 0.00	54.00 ± 0.00	48.79 ± 1.50
Intensive care	70.38 ± 14.22	49.63 ± 7.00	52.45 ± 9.12
Oncology	62.77 ± 12.75	46.50 ± 7.11	54.70 ± 1.50
***F* (*p*)**	4.181 (0.001)*	2.075 (0.069)	1.739 (0.126)
**Number of hours worked per week**			
20– < 40	65.00 ± 1.10	52.13 ± 8.19	61.20 ± 6.36
≥ 40	64.17 ± 13.35	46.23 ± 7.20	54.42 ± 9.33
***t* (*p*)**	2.128 (0.121)	12.267 (< 0.001)*	1.493 (0.227)
**Previous information of tecnonology training**			
Yes	65.84 ± 13.17	47.96 ± 7.88	52.39 ± 9.26
No	60.37 ± +14.48	46.42 ± 7.28	57.11 ± 9.41
***t* (*p*)**	3.126 (0.002)*	1.595 (0.112)	3.981 (< 0.001)*
**Degree of familiarity with IT system**			
Familiar	61.82 ± 15.03	48.73 ± 7.51	54.80 ± 10.11
Slightly Familiar	64.81 ± 12.26	46.15 ± 6.42	54.98 ± 9.13
Not familiar at all	71.00 ± 8.36	38.20 ± 8.19	49.58 ± 4.33
***F* (*p*)**	3.663 (0.027)*	15.971 (< 0.001)*	2.159 (0.118)

*Note:* The bold values indicate statistical significance. An asterisk (*) denotes statistically significant *p*‐values at *p* ≤ 0.05.

Abbreviations: *F*, *F* for One way ANOVA test; *p* value for comparing between/among the studied categories; SD, Standard deviation; *t*, Student *t*‐test.

## 4 Discussion

Grounded in the transaction‐based stress model (Lazarus and Folkman 1984), this study examined the dual impacts of technostress creators and inhibitors on nurses' psychological and work outcomes. The study findings support all three hypotheses, demonstrating that technostress creators negatively affect job satisfaction and commitment while increasing strain, whereas technostress inhibitors serve as protective factors that buffer these negative effects. Techno‐uncertainty emerged as the most significant stressor, while literacy facilitation and technical support were identified as the most effective organisational supports. This discussion section interprets these findings in light of existing theory and research, explores unexpected patterns and considers demographic variations in technostress experiences.

### 4.1 Descriptive Levels of Technostress, Inhibitors and Outcomes

The study identified moderate levels of technostress creators among nurses, with techno‐uncertainty emerging as the most significant stressor. This likely reflects the frequent updates and evolving nature of healthcare digital systems, which demand continuous learning and adaptation. Such changes introduce ambiguity and unpredictability, thereby intensifying stress levels. Ragu‐Nathan et al. (2008) and Califf et al. (2020) similarly observed that system instability and unclear expectations exacerbate user strain. Supporting these findings, Shaban et al. (2025) reported that nurses experience moderate levels of technostress, largely due to the dual pressures of adapting to new technologies while maintaining high standards of patient care—factors that contribute to frustration, feelings of inadequacy and fear of professional obsolescence. Huter et al. (2020) further emphasised that the need for nurses to remain proficient in a wide array of medical technologies adds to technostress in complex clinical environments.

In contrast, technostress inhibitors were positively perceived by nurses, indicating that organisational mechanisms, particularly literacy facilitation and technical support, play a crucial role in mitigating digital stress. Access to sufficient training and prompt support seems to improve nurses' perception of their control over technology. Supporting this, Stadin et al. (2020) found that digital literacy programs and dependable technical support serve as effective buffers against technostress, alleviating strain and reinforcing user confidence in technological systems. Recent evidence further supports this notion: Abou Hashish, Alsayed, et al. (2025) and Abou Hashish and Alnajjar (2024, 2025) found that enhancing digital proficiency encompassing knowledge, attitudes and skills significantly influences nursing readiness and engagement for technology integration.

This pattern of results is echoed in international studies. Brandon (2024) similarly identified techno‐uncertainty as the most prominent technostress factor among nurses, alongside moderate to high levels of technostress inhibitors—particularly training and involvement facilitation—as key stress‐reducing supports. Keshavarz et al. (2025) reported that 41% of healthcare professionals experienced moderate technostress, with techno‐uncertainty emerging as the leading contributor. Likewise, Golz et al. (2024) highlighted technostress as a growing global concern, stressing the dual need to reduce stress‐inducing factors and strengthen organisational support to safeguard healthcare workforce well‐being.

### 4.2 Relationship Among Technostress Variables

Correlation and regression analyses confirmed significant links between technostress and nurses' work outcomes, providing empirical support for Hypotheses 1 and 2. Technostress creators—especially techno‐overload and techno‐uncertainty—were strong negative predictors of job satisfaction, organisational and continuance commitment, and positively associated with strain. These results indicate that constant digital demands without sufficient support contribute to psychological distress and disengagement. Supporting this, Golz et al. (2024) and Wirth et al. (2024) reported that technostress increases emotional exhaustion and reduces job satisfaction. Marchiori et al. (2020) found a direct negative effect on satisfaction and an indirect effect on commitment. Shaban et al. (2025) linked technostress to burnout and lower emotional intelligence.

In contrast, technostress inhibitors—especially literacy facilitation, technical support and involvement—were positively associated with job satisfaction and commitment, and negatively with strain, underscoring their protective role. When nurses feel digitally competent and supported, technostress is reduced, enhancing professional engagement. These findings align with Galiano et al. (2024), who reported that IT training and support lowered perceived workload and improved well‐being. Similarly, Stadin et al. (2020) found that digital literacy and organisational support help buffer the adverse effects of technostress. Recent evidence from Abou Hashish, Khattab, et al. (2025) reinforces this interpretation, demonstrating that organisational support and knowledge management practices significantly influence evidence‐based practice implementation—suggesting that supportive organisational mechanisms facilitate not only technology adoption but also broader professional capabilities among nurses.

The mediation analysis (Hypothesis 3) revealed that technostress inhibitors partially mediate the relationships between technostress creators and work outcomes, demonstrating a buffering effect consistent with the transaction‐based model's stress‐buffering hypothesis. This finding aligns with Pansini et al. (2023), who noted that technostress may not lead to negative outcomes when balanced by support and coping strategies. Similarly, Gaudioso et al. (2017) and Consiglio et al. (2023) emphasised that workplace resources can buffer the psychological impact of techno‐stressors, with burnout acting as a key mediator. Nascimento et al. (2025) found that IT mindfulness and coping flexibility enhance job satisfaction and performance. Tarafdar et al. (2019) further distinguished between harmful techno‐distress and motivating techno‐eustress, depending on the presence of enabling structures.

### 4.3 Nurses' Demographic Characteristics and Technostress Variables

The study revealed several significant associations between nurses' demographics and their technostress experiences, suggesting that individual differences moderate the stress appraisal process as proposed by the transaction‐based model. Female and Saudi nurses reported higher exposure to technostress creators, possibly due to heavier involvement in electronic documentation and administrative duties, as well as cultural and digital exposure differences. These findings align with Wirth et al. (2024), Shan et al. (2023) and Romem and Rozani (2024), who reported increased technostress among female nurses due to multitasking and broader digital responsibilities.

Moreover, younger nurses (20–< 30 years) experienced more technostress creators and perceived fewer inhibitors, likely reflecting limited clinical experience and reduced confidence in digital system use. Huter et al. (2020) similarly observed that younger healthcare workers, while tech‐savvy, often lack contextual skills to apply digital tools effectively under pressure. This finding is theoretically significant because it challenges the common assumption that younger, digitally native workers experience less technology‐related stress. Instead, the results suggest that technological familiarity does not automatically translate to reduced technostress in high‐stakes clinical contexts. The transaction‐based model helps explain this paradox: younger nurses may possess strong technical skills (resources), but when faced with complex clinical scenarios requiring integration of technology with patient care judgement (high demands), their limited clinical experience creates a resource‐demand mismatch that intensifies stress.

Nurses with master's degrees reported the highest technostress levels, likely due to expanded roles involving leadership and advanced data use. Golz et al. (2024) supported this, linking leadership roles with greater digital responsibility and stress. Work area was another key factor. ICU nurses experienced the highest technostress due to constant interaction with advanced monitoring systems and electronic records. This aligns with findings from Keshavarz et al. (2025), Shaban et al. (2025) and Elsyed et al. (2020), who linked high‐acuity units to greater digital strain due to workload, infrastructure and training differences.

Interestingly, nurses with fewer working hours perceived more technostress inhibitors, possibly because a lighter workload allows greater access to support and reflection. Stadin et al. (2020) and Alsayed et al. (2022) similarly noted that reduced workloads enable proactive coping and improve well‐being. This finding supports the transaction‐based model's emphasis on resource availability: when nurses have sufficient time (a key resource), they can better access and utilise organisational support systems. Nevertheless, some studies report mixed findings. Alobayli et al. (2023) identified demographic and contextual influences on EHR‐related stress, while Brandon (2024) found no significant association, suggesting variability depending on organisational context and technology type.

### 4.4 Theoretical Interpretation

The study findings align with the transaction‐based stress model (Lazarus and Folkman 1984) in several important ways. The prominence of techno‐uncertainty as the leading stressor reflects the model's emphasis on unpredictability and ambiguity as core determinants of stress appraisal. When nurses cannot anticipate system changes or feel uncertain about their ability to master new technologies, they tend to engage in threat‐based primary appraisal, which increases psychological strain. This interpretation corresponds with Tarafdar et al. (2019), who differentiated between techno‐distress when demands exceed resources and techno‐eustress when demands are perceived as manageable challenges.

Moderate levels of technostress creators combined with relatively high levels of inhibitors indicate a state of managed technostress, where digital stressors exist but remain controlled due to supportive organisational conditions. This fits with the stress‐buffering mechanism implied in the transaction‐based model, suggesting that positive situational resources can modify the relationship between stressors and strain responses.

The findings contribute to technostress theory within healthcare environments. The strong association between technostress creators and negative work outcomes mirrors earlier findings in nonclinical settings, highlighting the applicability of this framework to healthcare despite clinical complexity. The stronger‐than‐expected protective role of inhibitors indicates that organisational support may hold greater influence in healthcare, where technology breakdowns carry direct implications for patient safety. This perspective aligns with the concept of consequences salience, in which errors have highly visible and immediate outcomes that heighten the importance of digital competence.

This interpretation is consistent with Abou Hashish, Alsayed, et al. (2025), and Abou Hashish and Alnajjar (2025) who noted that digital empathy depends on both competent technology use and supportive organisational environments. The partial mediation observed suggests that although organisational support is vital, reducing technological demands remains necessary. This supports a dual‐path approach consistent with Conservation of Resources theory (Hobfoll 1989), which emphasises that both resource depletion and resource gains independently affect well‐being. The findings also reinforce recent developments in understanding technology‐related psychological burden in nursing. Abou Hashish and Alnajjar (2025) emphasised that digital resilience, promoted by supportive organisational environments, may similarly protect nurses from technology‐induced strain and represents a valuable direction for further research.

### 4.5 Strengths and Limitations

A major strength of this study is its rigorous theoretical grounding in the transaction‐based stress model and comprehensive analysis of technostress using validated instruments and advanced statistical methods. The use of structural equation modelling (SEM) enabled deeper insights into both direct and mediated relationships between technostress factors and work outcomes, providing empirical validation of the stress‐buffering hypothesis. Furthermore, the study advances nursing science by integrating technostress with emerging concepts such as digital compassion fatigue and digital resilience. However, the cross‐sectional design limits causal interpretation, and reliance on self‐reported data may introduce common method bias. The use of a convenience sample from a single Saudi hospital also restricts the generalizability of findings to other settings, especially those with differing resources or cultural contexts. Additionally, while the study provides valuable insights into technostress at one point in time, longitudinal research is needed to examine how technostress evolves as nurses gain experience with digital systems. These limitations underscore the need for broader, mixed‐methods and multi‐site research.

## 5 Conclusion

Grounded in the transaction‐based stress model, this study provides empirical validation of how technostress affects nurses' psychological well‐being and work outcomes through both direct and buffered pathways. All three sets of hypotheses were supported, confirming that technostress creators, particularly techno uncertainty and techno overload, significantly predict increased strain and reduced job satisfaction, organisational commitment and continuance commitment. Conversely, technostress inhibitors, including digital literacy facilitation, technical support and participatory involvement, not only promote positive work experiences directly but also buffer the adverse effects of technostress creators, validating the stress buffering hypothesis central to the transaction‐based model. Demographic characteristics further moderate these relationships, with younger nurses, female nurses, and those in high‐acuity units experiencing higher technostress exposure. These findings extend the technostress literature by demonstrating its applicability to healthcare contexts and by introducing the concept of digital resilience as a protective factor. Healthcare organisations must implement comprehensive, theory‐driven strategies that address both demand reduction, minimising technostress creators and resource enhancement, strengthening technostress inhibitors, to sustain workforce well‐being in increasingly digital care environments.

## 6 Implications for Practice, Management, Policy and Future Research

The findings of this study underscore the need for a comprehensive approach to managing technostress among nurses, with implications for clinical practice, healthcare leadership and ongoing research.

*For nursing practice*, tailored training programs should be developed to enhance nurses' digital competencies, particularly among younger or less experienced staff who may be more vulnerable to techno‐uncertainty. Creating a culture of continuous learning, psychological safety and peer support in the use of digital systems can empower nurses to navigate complex technologies with greater confidence. Equipping nurses with practical skills in digital problem‐solving and resilience‐building is essential to sustaining performance in increasingly tech‐driven care environments.

*For healthcare and nursing management*, proactive organisational policies must prioritise technostress mitigation. This includes providing real‐time technical support, ensuring equitable access to digital training across departments and demographic groups, and engaging nurses in the design, implementation and evaluation of digital tools. Flexible scheduling, workload balancing and intuitive system design can reduce digital fatigue and foster higher system usability. Furthermore, leaders should consider incorporating broader digital transformation strategies—such as promoting AI literacy and strengthening nursing informatics leadership—as part of their workforce development plans. By empowering nurses to take part in digital governance and innovation, healthcare systems can enhance staff engagement, technology acceptance and overall care quality.

*For future research*, longitudinal and mixed‐method designs are recommended to capture the dynamic and evolving nature of technostress in healthcare settings. Qualitative investigations may uncover contextual nuances and coping strategies that are not readily accessible through surveys. Additionally, examining moderating variables such as resilience, digital self‐efficacy and leadership styles may provide further insights into how technostress can be managed or prevented. Expanding research across diverse cultural and institutional settings is also vital to understanding how contextual factors influence technostress and its outcomes. Finally, research should investigate the temporal dynamics of technostress—particularly whether the relationship between ICT training and technostress follows a curvilinear pattern as nurses progress from novice to expert users. Finally, integrating technostress research with emerging concepts such as digital compassion fatigue and digital empathy may provide a more holistic understanding of how technology affects the psychological and relational dimensions of nursing practice.

## Author Contributions

Both authors (E.A.A.H., H.A.A.) have substantial contributions to the manuscript. Study design: E.A.A.H., H.A.A. Data collection: E.A.A.H., H.A.A. Data analysis: E.A.A.H., H.A.A. Study supervision: E.A.A.H. Manuscript writing: E.A.A.H., H.A.A. Critical revisions for important intellectual content and correspondence: E.A.A.H.

## Funding

The authors have nothing to report.

## Ethics Statement

The study follows international ethical principles of the Helsinki Declaration. King Abdullah International Medical Research Center (KAIMRC) Institutional Review Board (IRB) approved the study with IRB approval no. NRJ24/032/8.

## Consent

Informed consent was obtained from all subjects.

## Conflicts of Interest

The authors declare no conflicts of interest.

## Supporting information


**Table S1:** Correlation among technostress creators and technostress inhibitors and technostress outcomes among nurses.

## Data Availability

The data that support the findings of this study are available from the corresponding author upon reasonable request.
