# Supplementary material for: Technostress Creators and Inhibitors: Dual Impacts on Nurses' Psychological and Work Outcomes
Source: Nurs Open. 2026 Mar 31;13(4):e70458. doi: 10.1002/nop2.70458 (PMC13140389; doi:10.1002/nop2.70458)
Supplement: Supplementary file 1 — Table S1: Correlation among technostress creators and technostress inhibitors and technostress outcomes among nurses. [file NOP2-13-e70458-s001.docx]

**Table S1.** *Correlation among technostress creators and technostress inhibitors and technostress outcomes among nurses*

| **Variables** |  | **TC**  **creators** | **TS**  **Inhibitors** | **Strain** | **Job Satisfaction** | **Organizational commitment** | **Continuance commitment** |
| --- | --- | --- | --- | --- | --- | --- | --- |
| Technostress creators | r |  |  |  |  |  |  |
|  | p | - |  |  |  |  |  |
|  | p | <0.001* |  |  |  |  |  |
| Technostress inhibitors | r | -0.259 | - |  |  |  |  |
|  | p | <0.001* |  |  |  |  |  |
| Strain | r | 0.277 | -0.885 |  |  |  |  |
|  | p | <0.001* | <0.001* |  |  |  |  |
| Job Satisfaction | r | -0.206 | 0.154 | -0.194 |  |  |  |
|  | p | 0.001* | 0.015* | 0.002* |  |  |  |
| Organizational commitment | r | -0.214 | 0.396 | -0.402 | 0.430 |  |  |
|  | p | 0.001* | <0.001* | <0.001* | <0.001* |  |  |
| Continuance commitment | r | -0.302 | 0.349 | -0.386 | 0.289 | 0.456 |  |
|  | p | <0.001* | <0.001* | <0.001* | <0.001* | <0.001* |  |
| Technostress outcomes | r | -0.343 | 0.367 | -0.354 | 0.426 | 0.545 | 0.378 |
|  | p | <0.001* | <0.001* | <0.001* | <0.001* | <0.001* | <0.001* |

*r: Pearson coefficient *: Statistically significant at p ≤ 0.05*
